# Paternal impact on the life course development of obesity and type 2 diabetes in the offspring

**DOI:** 10.1007/s00125-019-4919-9

**Published:** 2019-08-27

**Authors:** Gemma C. Sharp, Debbie A. Lawlor

**Affiliations:** 1 MRC Integrative Epidemiology Unit, University of Bristol, Oakfield House, Oakfield Grove, Bristol, BS8 2BN UK; 2 Bristol Dental School, University of Bristol, Bristol, UK; 3 Population Health Science, Bristol Medical School, University of Bristol, Bristol, UK; 4 NIHR Bristol Biomedical Research Centre, University Hospitals Bristol NHS Foundation Trust and the University of Bristol, Bristol, UK

**Keywords:** Developmental origins of health and disease, Epigenetics, Fathers, Life course development, Obesity, Paternal effects, Review, Type 2 diabetes

## Abstract

The aetiologies of obesity and type 2 diabetes are incredibly complex, but the potential role of paternal influences remains relatively understudied. A better understanding of paternal influences on offspring risk of obesity and type 2 diabetes could have profound implications for public health, clinical practice and society. In this review, we outline potential biological and social mechanisms through which fathers might exert an impact on the health of their offspring. We also present a systematically compiled overview of the current evidence linking paternal factors to offspring development of obesity and type 2 diabetes throughout the life course. Although evidence is accumulating to support paternal associations with offspring outcomes, more high-quality research is needed to overcome specific methodological challenges and provide stronger causal evidence.

**Electronic supplementary material:**

The online version of this article (10.1007/s00125-019-4919-9) contains peer-reviewed but unedited supplementary material including a slideset of the figures for download, which is available to authorised users.


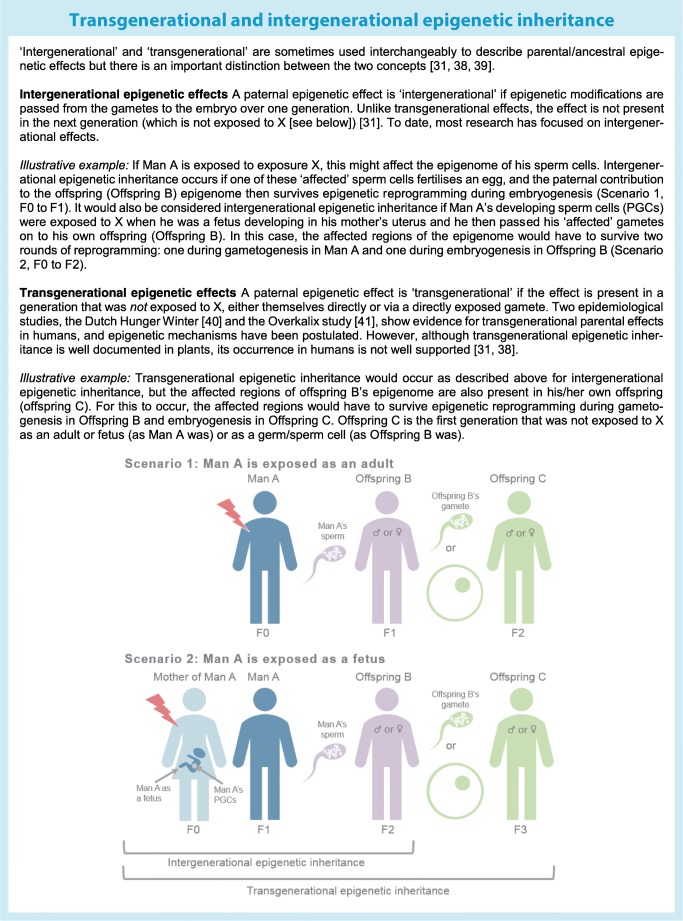


## Introduction

Research on the early-life origins of health and disease has traditionally focused on the maternal (mostly intrauterine) impact on offspring health [1, 2]. Although much of this work has been correlative, it has also produced some causal evidence that certain maternal characteristics (for example, gestational diabetes and extreme obesity in pregnancy) influence offspring greater adiposity and risk of type 2 diabetes [3]. Because of this, it has been suggested that overweight mothers might be the root cause of the current obesity epidemic [4]. However, the aetiologies of obesity and type 2 diabetes are incredibly complex and there are increasing calls for more research on other causal factors, including postnatal and paternal influences [5, 6].

In this review, we discuss the implications of paternal impacts on offspring risk of obesity and type 2 diabetes. We then outline potential biological and social mechanisms through which fathers might exert an impact on the health of their offspring, before providing an overview of the current evidence linking paternal factors to offspring development of obesity and type 2 diabetes throughout the life course. Finally, we discuss future research challenges and suggest potential strategies to overcome them.

## Paternal impacts on obesity and type 2 diabetes risk: implications for public health, clinical practice and society

Appreciation of the idea that fathers might influence risk of obesity and type 2 diabetes in their offspring would challenge assumptions about the causal primacy of maternal effects in pregnancy [2] and could have profound, wide-reaching implications.

### Implications for public health, policy and clinical practice

Fathers are a potential target for advice and interventions to improve offspring health, including risk of obesity and type 2 diabetes. However, currently very little health advice is offered to fathers-to-be. In a recent (2013) review of preconception care policy, guidelines and recommendations from six European countries, care was found to be inconsistent and fragmentary for healthy women and no country published specific guidelines for men alone [7]. Advice aimed at fathers-to-be has the potential to improve men’s health, maternal health and the health of their offspring,

There is considerable sociodemographic variation in paternal involvement during pregnancy and parenting [8], but a greater appreciation of the role of fathers in shaping offspring health across the life course could lead to efforts to increase involvement amongst groups who tend to be more distanced or disengaged. There is a large body of evidence suggesting that this would be beneficial for the health of the whole family [9–11]. A better understanding of paternal impacts could also translate into improved provision of paternity leave and strategies to support paternal childcare by employers.

### Implications for society

A greater understanding of the role of fathers could lessen the focus on mothers, and sometimes non-pregnant women of child-bearing age, as the main ‘vectors’ for the intergenerational transmission of disease risk [12]. As mentioned above, mothers have been ‘blamed’ for the obesity epidemic in the scientific literature [4], and this has been widely covered in the mainstream media too [13, 14]. There are multiple examples of these ideas infiltrating the public discourse around maternal impacts on offspring health in a way that can limit women’s autonomy, increase surveillance and lead to social reproach and even criminalisation [2, 15]. At the same time, these ideas diminish the role fathers play in the care of their children and downplay the role of wider societal structures that influence health and wellbeing. Effective translation of research on paternal impacts on offspring health could help to balance the public perception of intergenerational harms and lessen the burden on pregnant women.

## Potential mechanisms for paternal impacts on offspring health

**Fig. 1:**
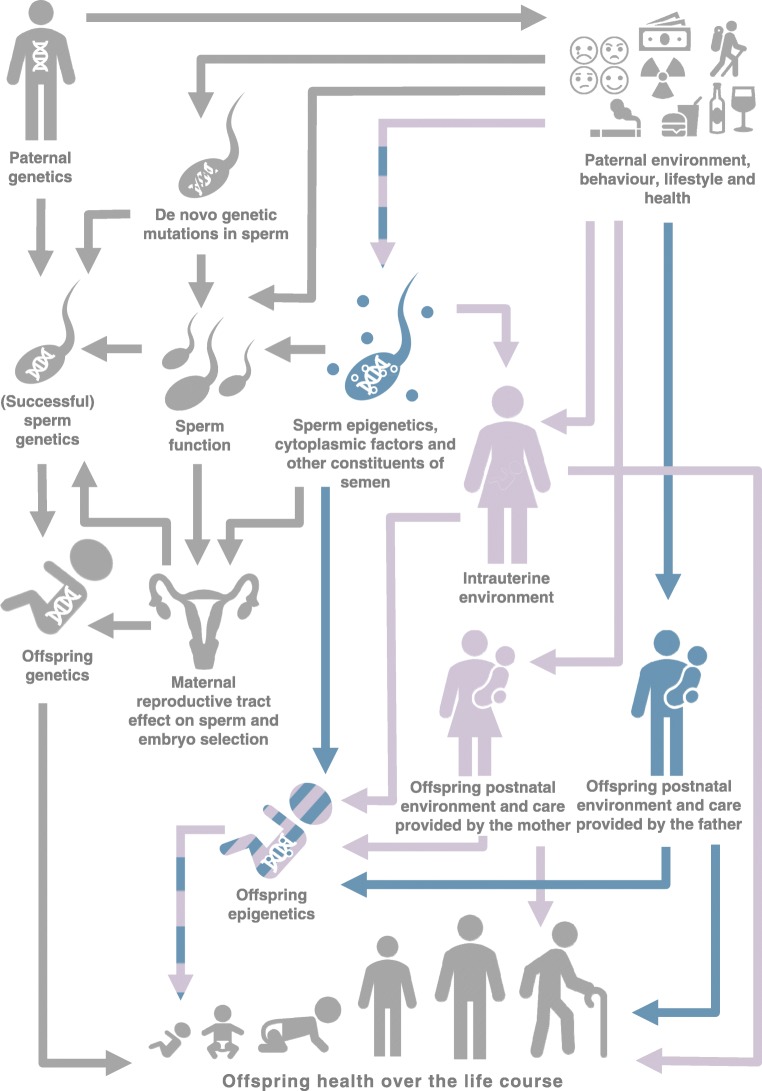
Potential mechanism through which fathers might have an impact on offspring health, including obesity and risk of type 2 diabetes. Pathways for ‘true’ paternal effects are highlighted in blue (direct paternal impact) and purple (indirect paternal impact). Genetic pathways (which are not considered ‘true’ paternal effects [19]) are shown in grey. For simplicity, not all potential connections are depicted here (e.g. we have not drawn a connection from offspring genetics to offspring epigenetics, although one exists). This figure is available as part of a downloadable slideset

There are several potential mechanisms through which fathers might influence health and risk of obesity or type 2 diabetes in their offspring (reviewed in detail in [16, 17]). In evolutionary biology, the term ‘paternal effect’ (and ‘maternal effect’ [18]) has a precise definition describing a causal association between variation in paternal (or maternal) genotype or phenotype and variation in offspring phenotype, independently of variation in offspring genotype [19]. In this paper, we talk more broadly about paternal impacts or influences, which do not necessarily conform to these stricter definitions of paternal effects. We categorise the potential mechanisms underlying paternal impacts as genetic or non-genetic, and direct or indirect (see Fig. 1); however, there is likely to be a high-degree of overlap and interaction between the various pathways we describe.

### Direct genetic paternal impacts

Through genetic transmission of alleles, fathers provide roughly half of their offspring’s nuclear DNA, thereby influencing their genetic risk of disease. This includes offspring inheritance of genetic information at paternally derived imprinted loci, where only the paternal allele is expressed because the maternal allele is silenced by DNA methylation [20]. However, there is evidence that factors like paternal age and environmental exposures can also directly influence offspring genotype and subsequent phenotype through inducing DNA damage and de novo genetic mutations in the male germline [21–23]. Another plausible explanation is that the paternal environment selects for certain haploid genomes by skewing the genotype distribution in ejaculate and/or by causing mutations that influence sperm function and chances of success [16]. For example, high-fat-diet-induced obesity can reduce sperm number, motility, morphology and reduce capacitation and oocyte binding in mice [24].

Although these genetic effects could underlie a paternal impact on offspring phenotype, it should be noted that they are not considered true paternal effects because variation in offspring phenotype would reflect variation in offspring genotype; i.e. true paternal effects are those that occur independently of offspring genotype [19].

### Direct non-genetic paternal impacts

In species exhibiting paternal care (including humans), perhaps the most obvious way that fathers can influence offspring phenotype directly is through a direct effect on the offspring postnatal environment**.** This will be affected by multiple factors, including the father’s frequency and style of parenting, their socioeconomic and cultural circumstances and their own health behaviours [25–27]. In some cases, epigenetic mediation might be involved in the biological manifestation of these effects [28]. There is also growing evidence that direct non-genetic paternal effects can be transmitted prenatally. The most commonly postulated mechanism is transmission of epigenetic factors in sperm, such as DNA methylation, chromatin modifications and non-coding RNAs [29]. We consider a broad definition of the term ‘epigenetics’ as heritable molecular changes that affect gene expression but do not involve changes to the underlying sequence of DNA [30]. DNA methylation is the most widely studied epigenetic mechanism in humans in this context. In mammals, DNA methylation undergoes two rounds of ‘reprogramming’ between generations, which involves waves of demethylation and remethylation. The first round occurs shortly after fertilisation, during embryogenesis, and the second occurs in the primordial germ cells (PGCs) of the developing fetus during gametogenesis. For DNA methylation patterns to be passed between generations to influence gene expression and phenotype, some loci must escape this reprogramming. The number of reprogramming waves that must be overcome depends crucially on whether the inheritance is ‘intergenerational’ or ‘transgenerational’ [31] (see Text box). There is evidence that methylation at some loci of the sperm epigenome can escape epigenetic reprogramming at embryogenesis. Although the majority of the sperm genome is packaged in protamines, the small fraction (5–10%) that remains attached to histones may be protected from demethylation [32]. This fraction includes paternally derived imprinted loci [32, 33] . Proposed windows of susceptibility [34] when the gamete epigenome of a father-to-be might be particularly susceptible to environmental exposures are: (1) during PGC reprogramming in utero; (2) before puberty, as methylation is re-established; and (3) during each reproductive cycle, as methylation profiles become fully established in mature spermatozoa [34]. It has also been suggested that methylation at imprinted genes (because only one allele is active) might be particularly vulnerable to environmental perturbation [35]. Aside from DNA methylation, there is increasing evidence that sperm RNAs and histone modifications are compelling candidates for epigenetic inheritance, but understanding of these mechanisms is in its infancy [32].

In addition to epigenetic factors, sperm can also transmit cytoplasmic factors to offspring at fertilisation. These include activation factor, centrosomes, messenger RNA and microRNAs, which could modify post-fertilisation events and offspring embryonic development [17, 23]. Components of the non-sperm fraction of the ejaculate (e.g. seminal fluid proteins, peptides, lipids, salts, etc.) have also been shown to vary with paternal environment [36] and there is some evidence from animals that, in addition to an effect on sperm function, success and selection, they can also have a direct effect on offspring phenotype [37], although the mechanisms are unclear.

### Indirect genetic paternal impacts

Indirect genetic paternal effects occur when the offspring phenotype is affected by genes being expressed in the father [42]. Paternal genetic liability to non-genetic factors, for example, health behaviours, like smoking, or certain epigenetic signatures, might influence offspring phenotype via ‘genetic nurture’, regardless of genetic transmission of the liability-increasing alleles. For example, Kong et al [43] showed that polygenic scores generated using only non-transmitted alleles for educational attainment were robustly associated with offspring educational attainment. The authors found similar effects estimates for polygenic scores generated using maternal and paternal non-transmitted alleles.

### Indirect non-genetic paternal impacts

Many pathways of influence between fathers and offspring are mediated or moderated by maternal responses, thus blurring the lines between maternally mediated paternal effects and male-induced maternal effects [19]. For example, seminal fluid can interact with female tract physiology with implications for embryo development [44]. In addition, female tract physiology (including any way in which it is modified by seminal fluid) influences sperm selection and, therefore, the ‘successful’ sperm and offspring genomes. During pregnancy, a father can influence a mother’s environment and physiology (e.g. through passive smoke exposure, stress, emotional support, etc), which might have an intrauterine environmental effect on the fetus (potentially via the fetal epigenome [45]). If this father-induced intrauterine exposure has an effect soon after fertilisation, it could disrupt epigenetic reprogramming in the embryo [34]. Additionally, a father can also influence the mother’s postnatal environment, which could affect (positively or negatively) the type and level of care she provides for offspring. Across the life course (both pre- and postnatally) there is also evidence that parents influence each other’s behaviours [46, 47], thereby further blurring the distinction between maternal and paternal impacts on offspring health.

## Literature review of paternal impacts on obesity and type 2 diabetes

Having outlined potential mechanisms for paternal impacts on offspring health, we now briefly assess the extent to which these have been studied in relation to paternal impacts on obesity and type 2 diabetes across the life course.

### Methods

To help ensure an unbiased review, we conducted a systematic search of PubMed using cross-sections of key words relating to paternity (‘paternal’, ‘father*’, ‘intergenerational’, ‘transgenerational’), and obesity (‘obes*’, ‘overweight’, ‘body weight’, ‘BMI’, ‘body mass index’, ‘fat’, ‘adiposity’, ‘adipose’) or type 2 diabetes (‘diabet*’, ‘T2D’, ‘metabol*’, ‘glucose’, ‘insulin’). We specified that these words had to be contained in the titles of identified articles. The search was conducted on the 13 March 2019 and identified 483 articles. After exclusion of non-journal articles (*n* = 27), reviews (*n* = 48) and papers not in the English language (*n* = 10), 398 abstracts were screened. Of these, 128 described original data on paternal exposures/characteristics and relevant (i.e. obesity- or type 2 diabetes-related) offspring outcomes. It should be noted that we did not consider studies where the directly exposed generation was female (see ‘Scenario 2’ illustration in the text box) to be studies of paternal exposures, even if the females were pregnant with male fetuses; we considered these to be studies of (grand-)maternal rather than paternal impacts. From the 128 included articles, data were extracted regarding the species studied, class of paternal exposure, class of offspring outcome and whether the study found evidence of a correlation. Information on the mechanism driving any potential paternal causal effect was also extracted from studies that explicitly investigated this. We did not formally assess the quality of included studies or conduct any meta-analyses. A full table of the studies included and our findings is provided in the electronic supplementary material (ESM) Table 1.

### Results

**Fig. 2:**
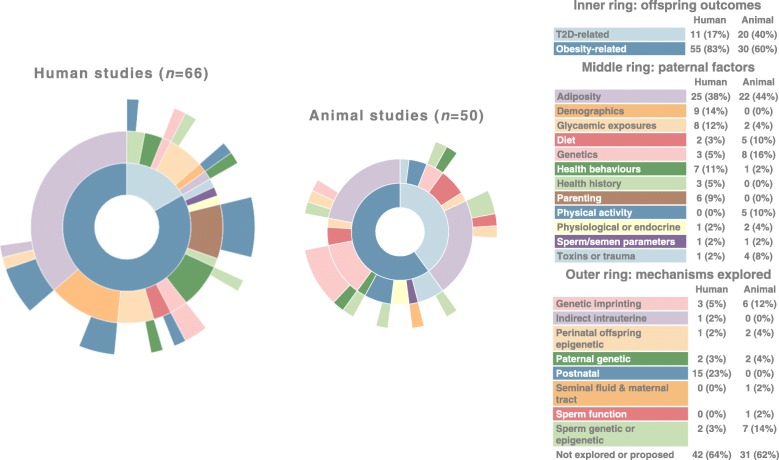
A summary of the outcomes, exposures and mechanisms explored by 116 studies that showed at least correlative evidence linking paternal characteristics to offspring risk of obesity and type 2 diabetes. The data used to produce this figure is provided in ESM Table 1. T2D, type 2 diabetes. This figure is available as part of a downloadable slideset

Of 128 studies, 78 (61%) were conducted in humans and 50 (39%) were conducted in animals (mainly rodents [*n* = 45], but also drosophila [*n* = 3], *Caenorhabditis elegans* [*n* = 1] and pigs [*n* = 1]). In 116 studies (91%), there was at least correlative evidence linking paternal factors to offspring obesity- or type 2 diabetes-related traits. All of the 12 null studies were of humans. Figure 2 summarises the focus of the 116 studies that found some evidence of association. Most of these (*n* = 47 [41%]) found a link between paternal adiposity (including BMI and high-fat-diet-induced obesity) and obesity- or type 2 diabetes-related offspring outcomes (including birthweight, body fat, BMI, obesity-related gene expression, adipose tissue remodelling, metabolic function, glucose tolerance and insulin sensitivity). Of the 12 null studies, two investigated paternal BMI and found no evidence of association with offspring obesity-related traits (i.e. 2/49 [4%] of studies of paternal adiposity were null). In addition to paternal adiposity, the other top five most studied paternal factors were genetic factors (11 non-null, 1 null), glycaemic exposures, such as diabetes and hyperglycaemia (10 non-null, 1 null), demographic factors such as socioeconomic position and age (9 non-null, 2 null), and health behaviours such as smoking and drinking alcohol (8 non-null, 1 null).

The majority of studies (*n* = 79 [62%]) did not explicitly explore potential mechanisms linking paternal factors to offspring obesity or type 2 diabetes. Of those that did, the most commonly explored mechanisms were postnatal (15 non-null, 5 null), genetic imprinting (9 non-null, 0 null), sperm epigenetics (7 non-null, 0 null), paternal genetics (4 non-null, 1 null) and perinatal offspring epigenetics (3 non-null, 0 null).

Only nine studies reported evidence of a paternal impact over more than two generations, suggesting a transgenerational effect. The vast majority of studies explored intergenerational associations only.

All the animal studies involved testing the effects of experimental manipulations, whereas all the human studies were based on observational data. One human study [48] was based on a randomised control trial of the effect of a weight loss intervention on fathers’ diet, but no offspring outcomes were explored in relation to whether the fathers were randomised to intervention or control. Results that were relevant to this review were cross-sectional baseline correlations between paternal and offspring obesogenic diet.

Animal studies appeared equally likely to explore potential mechanisms (19/50 [38%]) as human studies (30/78 [38%]), although this can be explained by 20 human studies where the exposure, and therefore the mechanism, was postnatal. Mechanisms involving genetics or prenatal factors were far more likely to be explored in animal studies (19/50, 38%) than human studies (10/78 [13%]).

### Discussion and suggestions for future research

In line with what we see in the developmental origins of health and disease field more widely [1, 2], compared with studies of maternal impacts, there have been relatively few studies of paternal impacts on offspring obesity and type 2 diabetes. Of the 128 studies that we identified, a high proportion showed evidence of at least a correlative link between paternal factors and these offspring outcomes across the life course (with a limited number of studies also exploring potential transgenerational effects), which highlights that this is a promising avenue of research that should be further pursued.

However, we also consider that a high proportion of non-null studies could indicate publication bias and/or be an artefact of our search strategy (i.e. we restricted our keyword searches to article titles, which are perhaps less likely to advertise null results). Our review aimed to provide an overview of the current literature on paternal impacts on offspring obesity and type 2 diabetes. Although beyond the scope of this paper, a more detailed review and meta-analysis to assess average effect estimates and evidence of heterogeneity and small-study (publication) bias is warranted. A previous systematic review and meta-analysis of human studies found only modest average effects of various paternal exposures on cardiometabolic outcomes and identified some issues with the quality of evidence [49].

Most of the studies we identified were human observational studies and causal evidence for paternal impacts (in humans at least) is lacking. Most studies did not explore the biological or social mechanisms through which fathers might influence obesity and type 2 diabetes risk in offspring, but identification and clarification of such mechanisms would be one way to strengthen causal inference.

There are some specific challenges to the study of paternal effects that must be tackled by future research. In human observational studies, two major challenges are confounding and bias. When studying paternal direct effects, a major source of confounding is likely to arise from high correlation with maternal exposures due to shared environments and assortative mating [50]. Compared with maternal data, paternal data may have a higher degree of measurement error (e.g. due to maternal-report rather than self-report) and missingness (partly due to birth cohort recruitment strategies that primarily target mothers and partly due to the overall lower response to health studies by men compared with women [2]). This could bias paternal estimates towards or away from the null depending on the nature of the error/bias, sample and study design [51]. We might expect high rates of non-paternity (whereby mothers are more genetically related to their children than fathers are) to have a similar biasing effect, but sensitivity analyses around this have shown that even with very high (and likely implausible) simulated rates of non-paternity, the bias was minimal [52]. Strategies to tackle these specific issues with confounding and bias include: (1) collecting more and better quality data on fathers or linking to other datasets, such as national registries, with more complete data on men; (2) triangulating evidence [53] from different causal inference techniques, such as Mendelian randomisation [54] and negative control designs [55]; and (3) testing the robustness of estimates to adjustments for simulated levels of non-paternity [50]. The same issues with observational data may not apply to animal experiments, but there are other issues, such as the focus on more extreme exposures than are usually seen in humans, and the translatability of some exposures and mechanisms to human contexts. We identified more human than animal studies of paternal exposures in our review, which is in line with our previous finding that the overwhelming focus on maternal exposures in the developmental origin of health and disease field is particularly evident in the animal literature [1].

## Conclusion

A better understanding of paternal influences on offspring risk of obesity and type 2 diabetes could have profound implications for public health, clinical practice and society. There are multiple possible mechanisms through which paternal exposures might influence offspring health and development. Evidence is accumulating to support paternal associations with offspring outcomes; however, more high quality research is needed to overcome specific methodological challenges and provide stronger causal evidence.

## Electronic supplementary material

Slideset of figures (PPTX 377 kb)

ESM Table (XLSX 52 kb)

## Data Availability

All data identified in our systematic review and used to support the conclusions of this paper are available in ESM Table 1.

## Abbreviation

PGC: Primordial germ cells
