# Supplementary material for: Paternal impact on the life course development of obesity and type 2 diabetes in the offspring
Source: Diabetologia. 2019 Aug 27;62(10):1802–10. doi: 10.1007/s00125-019-4919-9 (PMC6731203; doi:10.1007/s00125-019-4919-9)
Supplement: Supplementary file 1 — (PPTX 377 kb) [file 125_2019_4919_MOESM1_ESM.pptx]

## Slide 1
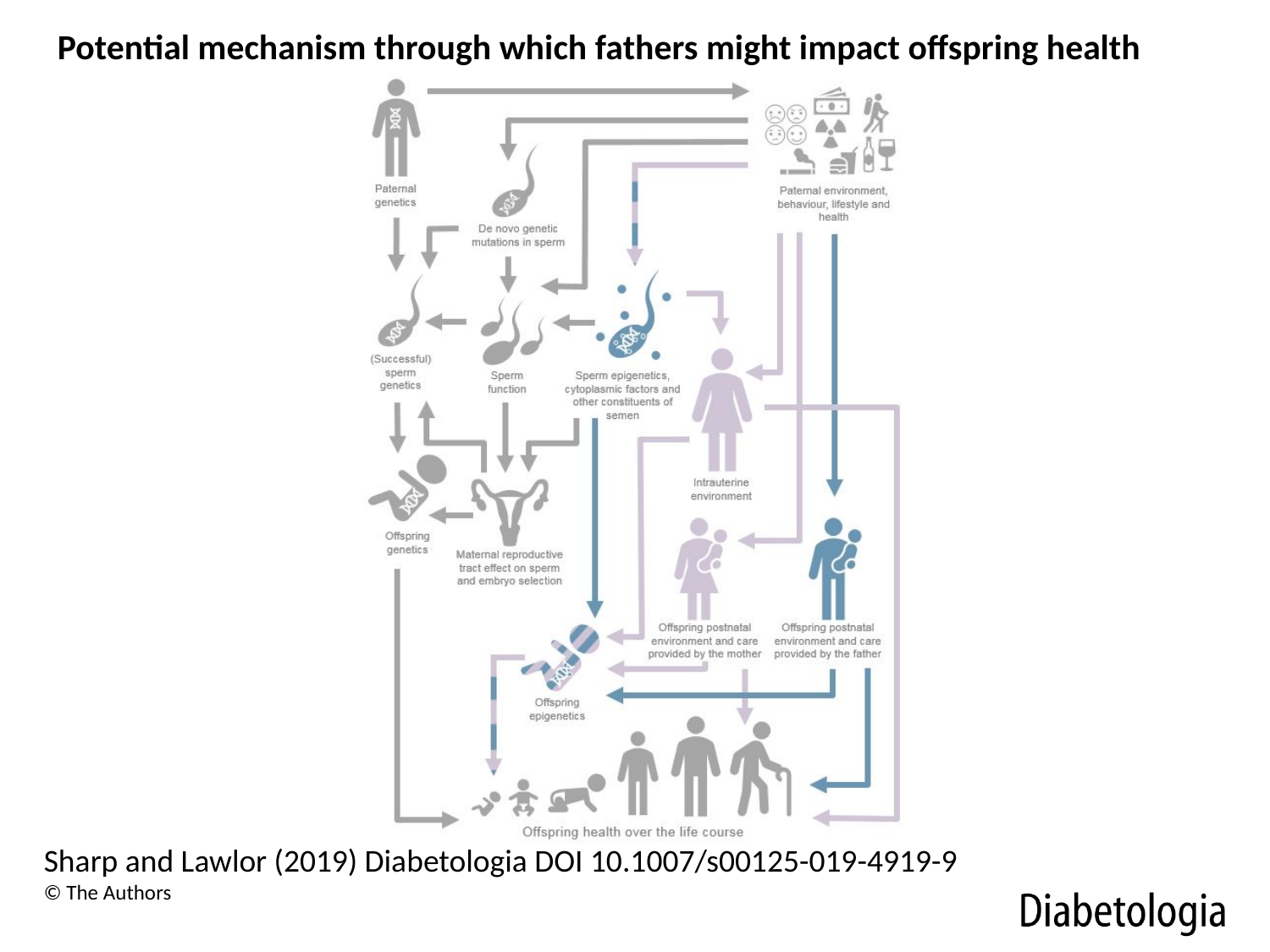

Potential mechanism through which fathers might impact offspring health
Sharp and Lawlor (2019) Diabetologia DOI 10.1007/s00125-019-4919-9
© The Authors

## Slide 2
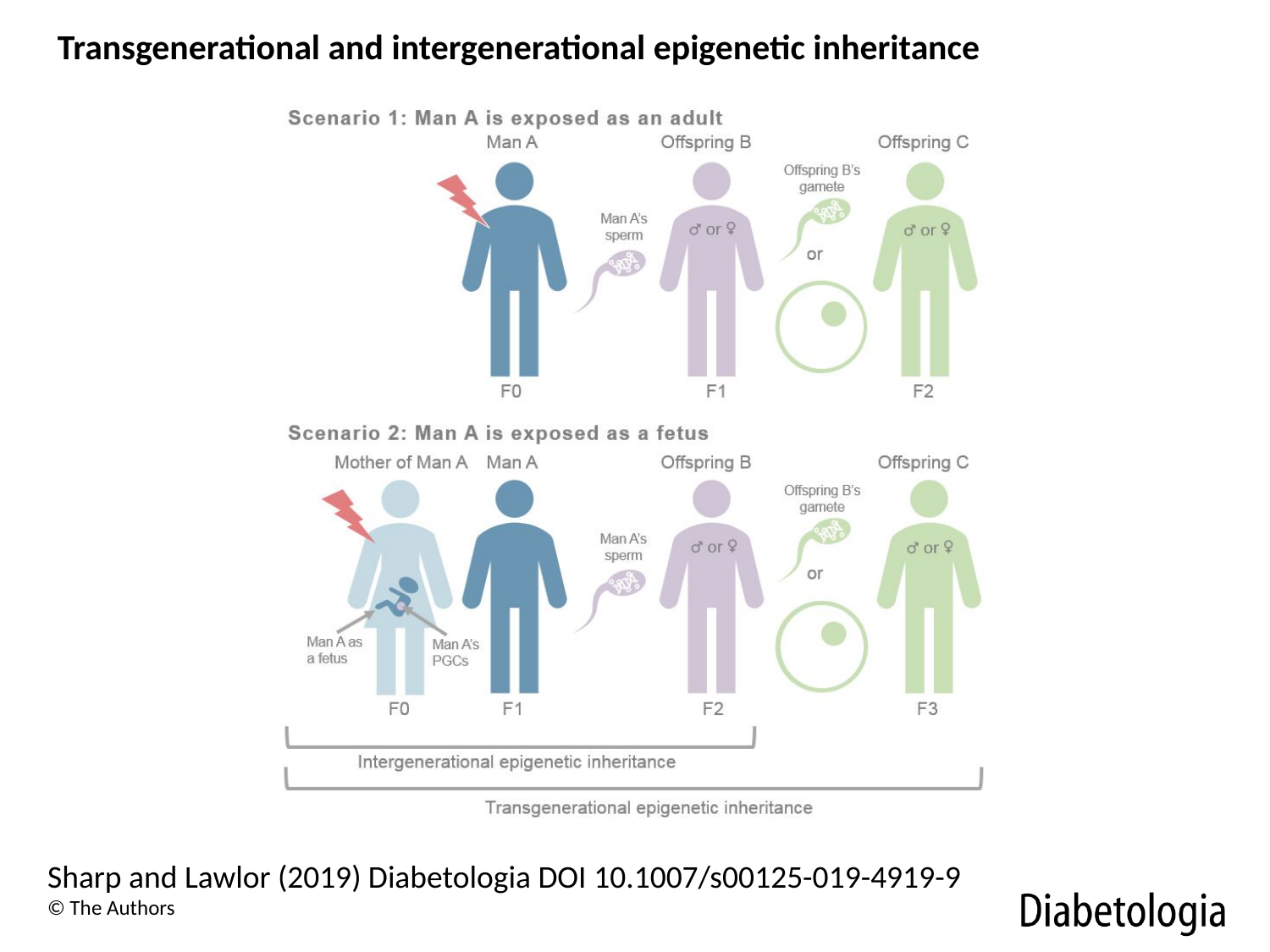

Transgenerational and intergenerational epigenetic inheritance
Sharp and Lawlor (2019) Diabetologia DOI 10.1007/s00125-019-4919-9
© The Authors

## Slide 3
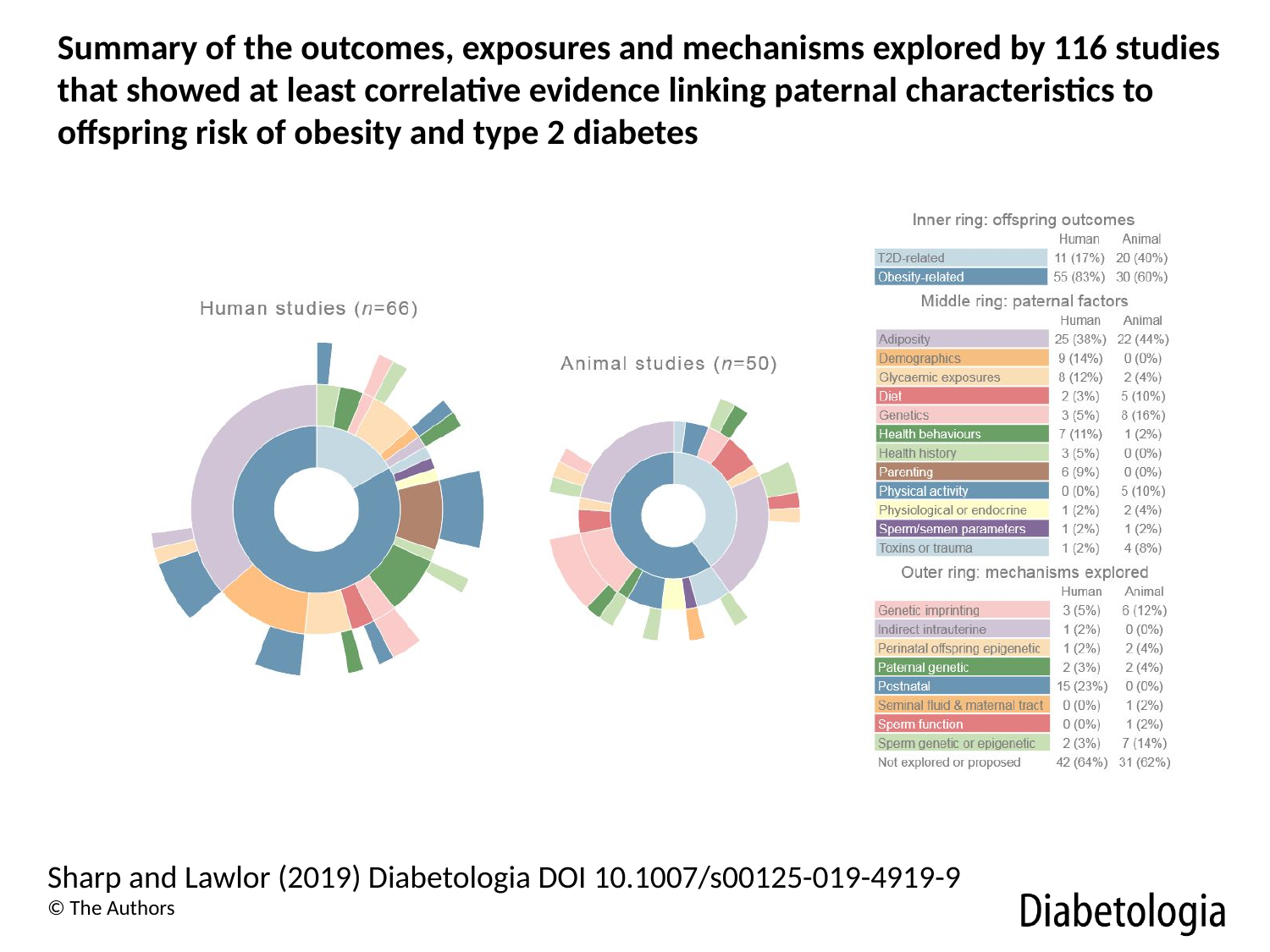

Summary of the outcomes, exposures and mechanisms explored by 116 studies that showed at least correlative evidence linking paternal characteristics to offspring risk of obesity and type 2 diabetes
Sharp and Lawlor (2019) Diabetologia DOI 10.1007/s00125-019-4919-9
© The Authors
